# The effect of squash domestication on a belowground tritrophic interaction

**DOI:** 10.1002/pei3.10071

**Published:** 2022-03-04

**Authors:** Charlyne Jaccard, Nicolas T. Marguier, Carla C. M. Arce, Pamela Bruno, Gaëtan Glauser, Ted C. J. Turlings, Betty Benrey

**Affiliations:** ^1^ Institute of Biology, Laboratory of Evolutionary Entomology University of Neuchâtel Neuchâtel Switzerland; ^2^ Laboratory of Fundamental and Applied Research in Chemical Ecology University of Neuchâtel Neuchâtel Switzerland; ^3^ Neuchâtel Platform of Analytical Chemistry University of Neuchâtel Neuchâtel Switzerland

**Keywords:** cucurbitacins, Diabrotica, domestication, predator, sequestration, squash

## Abstract

The domestication of plants has commonly resulted in the loss of plant defense metabolites, with important consequences for the plants' interactions with herbivores and their natural enemies. Squash domestication started 10′000 years ago and has led to the loss of cucurbitacins, which are highly toxic triterpenes. The banded cucumber beetle (*Diabrotica balteata*), a generalist herbivore, is adapted to feed on plants from the Cucurbitaceae and is known to sequester cucurbitacins, supposedly for its own defense. However, the evidence for this is inconclusive. In this study we tested the impact of squash domestication on the chemical protection of *D. balteata* larvae against a predatory rove beetle (*Dalotia coriaria*). We found that cucurbitacins do not defend the larvae against this common soil dwelling predator. In fact, *D. balteata* larvae were less attacked when they fed on cucurbitacin‐free roots of domesticated varieties compared to high‐cucurbitacin roots of wild plants. This study appears to be the first to look at the consequences of plant domestication on belowground tritrophic interactions. Our results challenge the generalized assumption that sequestered cucurbitacins protect this herbivore against natural enemies, and instead reveals an opposite effect that may be due to a tradeoff between coping with cucurbitacins and avoiding predation.

## 1 INTRODUCTION

For thousands of years, humans have imposed strong selection on domesticated crops. This has drastically altered crop phenotypes as compared to their wild relatives (Evans, 1993, Gepts, 2004). Domesticated plants have larger plant structures, are more nutritious and have greater yields under cultivation conditions. In addition, crop selection has reduced plant architectural complexity (Chen and Welter, 2005, Andow and Prokrym, 1990) and plant specialized metabolite diversity (Gols et al., 2008, Milla et al., 2015). Empirical evidence shows that domestication has decreased plant defenses against herbivores (Turcotte et al., 2014, Chen et al., 2015, Whitehead et al., 2017, Fernandez et al., 2021). Indeed, one of the most important consequences of plant domestication is the loss or reduction of plant specialized compounds that are toxic for herbivores (Meyer et al., 2012, Chen et al., 2015, Chacón‐Fuentes et al., 2015, Rodriguez‐Saona et al., 2011), therefore making plants more vulnerable to herbivory. For different crops, domestication has a direct impact on herbivore oviposition preferences (Idris and Grafius, 1996, Cardona et al., 1990, Bellota et al., 2013, Dávila‐Flores et al., 2013), growth (Cardona et al., 1990, Szczepaniec et al., 2013, Benrey et al., 1998), and survival (Cardona et al., 1990, Gols et al., 2008, Chen and Welter, 2005). Variation in host plant quality as a result of plant domestication will indirectly affect the third trophic level, such as predators and parasitoids that rely on the nutrients ingested by their victims (Chen et al., 2015, Chen et al., 2015). Effects on these natural enemies are expected to be particularly relevant for specialized insects that sequester defense compounds from their host plants, as these are often the types of compounds that are reduced in domesticated plants.

Studies that have investigated the effect of plant domestication on natural enemies show that, overall, they perform better on herbivores that feed on domesticated crops than on their wild counterparts (Benrey et al., 1998, Gols et al., 2008, Campan and Benrey, 2004, Harvey et al., 2011, Li et al., 2018). However, most of these studies have focused on aboveground herbivores and their associated natural enemies (βet al., 2015). Little is known about the impact of plant domestication on belowground plant‐insect interactions, particularly on how altered chemical traits in domesticated plants have influenced the susceptibility of belowground herbivores to soil predators.

One classical example of reduction in chemical defense as a result of crop domestication involves species of squash, *Cucurbita* spp.. There is ample knowledge about the history of domestication of *Cucurbita* (Smith, 1997, Nee, 1990, Barrera‐Redondo et al., 2021, Kates et al., 2021, Castellanos‐Morales et al., 2018), but the consequences of squash domestication for the interaction between herbivores and their natural enemies have hardly been explored. *Cucurbita* plants were domesticated during different independent events in the American continent (Sanjur et al., 2002, Kates et al., 2017, Chomicki et al., 2019). The oldest evidence for squash domestication dates to about 10,000 years ago in Mesoamerica along with maize and beans (Lira et al., 2016). One of the main traits altered during the domestication of squash is the loss of cucurbitacins in the different plant organs (Nee, 1990). These defense metabolites are oxygenated tetracyclic triterpenes that are extremely bitter and render plants toxic or unpalatable to many invertebrates and vertebrate herbivores, including humans (Da Costa and Jones, 1971, Ferguson and Metcalf, 1985). However, cucurbitacins can also serve as kairomones (semiochemicals that benefit certain receivers) (Metcalf, 1986) for a number of specialized phytophagous chrysomelid beetles of the Old World tribe Luperini, in the genus *Aulacophor*a (Jaccard et al., 2021, Chambliss and Jones, 1966, Castellanos‐Morales et al., 2018, Lewis and Metcalf, 1996). Both squash and Diabroticine beetles share origins in Mesoamerica and have coevolved over an extended period of time (Metcalf, 1979, Metcalf, 1986, Metcalf and Lampman, 1989). Earlier studies have shown that those beetles have overcome the chemical defense of *Cucurbita* via physiological adaptations to bitter and toxic cucurbitacins and use them as attractants and possibly as feeding stimulants (Metcalf, 1979). Moreover, it has been hypothesized that species of *Diabrotica* actively seek bitter squash plants to accumulate cucurbitacins in their body and use them as a defense against natural enemies (Howe et al., 1976, Ferguson and Metcalf, 1985). This hypothesis was first supported by Ferguson and Metcalf (1985), who showed that a significant proportion of laboratory‐reared adult beetles of *Diabrotica* species (*D. balteata, D. undecimpunctata howardi,* and *D. virgifera virgifera*) and of *Acalymma vittatum* fed on varieties of bitter squash, were rejected by Chinese praying mantis. Cucurbitacins were also shown to be efficient against pathogenic fungi that infect eggs and larvae of *Diabrotica undecimpunctata howardi* (Tallamy et al., 1998, Brusti & Barbercheck, 1992). However, the evidence that cucurbitacins effectively protect herbivores against natural enemies is scarce and has been challenged (Gould and Massey, 1984, Brusti and Barbercheck, 1992, Barbercheck, 1993, Barbercheck et al., 1995). Indeed, while several studies showed that herbivores can sequester cucurbitacins from their host plants, they did not find evidence for their protective role against a diverse array of natural enemies (arthropods, birds, mice, and toads) (Brusti & Barbercheck, 1992, Gould and Massey, 1984). For example, Barbercheck et al. (1995) found that infection by entomopathogenic nematodes was not different between rootworm larvae (*D. undecimpunctata howardi*) fed on bitter or non‐bitter squash, although the nematodes' fecundity was lower when their hosts fed on bitter squash compared to corn, peanuts, or non‐bitter squash. Other studies have mostly examined the role of herbivore‐sequestered cucurbitacins against natural enemies in adult beetles that feed on aboveground tissues, and/or their eggs. However, for most squash species, the content of cucurbitacins is higher in the roots than in aboveground tissues (Jaccard et al., 2021). Moreover, the earlier studies exclusively used domesticated varieties of cucurbits (either cucumber or squash) with varying levels of cucurbitacin but in general with very low levels of these compounds (Nee, 1990, Chomicki et al., 2019) as compared to their wild counterparts (Jaccard et al., unpublished data).

In this study, we first confirmed the hypothesis that cucurbitacins levels are reduced in the roots of domesticated *Cucurbita argyrosperma*, and next tested if this affects the susceptibility of a root larval‐herbivore to a generalist predator. To do this, we compared the preference and performance of a generalist soil predator (*Dalotia coriaria*, formerly known as *Atheta coriaria*) when offered larvae of *Diabrotica balteata* fed on roots of wild *C. argyrosperma* with expected high cucurbitacin content or on roots of related domesticated varieties where the cucurbitacins ought to be nearly absent.

## 2 MATERIAL AND METHODS

### 2.1 Plants

*Cucurbita argyrosperma,* known as “calabaza pipiana” is an important crop in local agricultural systems in Mexico (Sánchez‐de la Vega et al., 2018). The oldest evidence of its domestication is ~8600 years old from the Xihuatoxtla shelter, in the state of Guerrero (Ranere et al., 2009). Plants can be found in tropical and semi desertic regions from the Southeastern United States through Mexico and Northern Central America (Lira et al., 2016). This is a diverse species in form, color and size of its seeds and fruits (Lira‐Saade, 1995).

Fruits of two wild *C. argyrosperma* populations (Wild Umar [WU] and Wild Bacocho [WB]) were collected in the region of Puerto Escondido along the Pacific coast in the state of Oaxaca, Mexico in January 2018, and their seeds were harvested (WU: 15°92′49.2” N, 97°15′09.77”W and WB: 15°86′44.6”N, −97°08′11.4″). The weather conditions in this region are hot and humid with an average temperature of 27°C and 84% relative humidity. Seeds of domesticated varieties were purchased from the KCB‐Samen GmbH Bottmingen, Schweiz. We used four varieties that have been selected for two different purposes, two varieties selected for fruit consumption: Vera Cruz Pepita (FVP) and Silver Edge (FSE) and two varieties selected as ornamentals: Cushaw Tricolor (OCT) and Navajo calabacita (ONC). These varieties had been previously used in a related study in which we found differences in their cucurbitacin content (Jaccard et al. unpublished results).

Depending on the experiment, either one individual seed or seven seeds of domesticated varieties were germinated in one individual plastic pot filled with a mixture of soil: sand 70:30 (Einheitserde, Sinntal‐Altengronau, Germany). As wild seeds take longer to germinate, they were planted 10 days before the domesticated seeds. Also, wild seeds were subjected to a specific germination procedure to enhance their germination rate: seed coats were pierced, scratched on both sides and placed in groups of 10 in a square Petri dish with wet cotton for 1 week in an incubator at 28 °C degrees in the dark. Germinated wild seeds were transferred following the same procedure as seeds from domesticated varieties. Plants with two cotyledons and two fully developed leaves (15 day‐old) were used for all the experiments. All plants were grown under controlled conditions in a greenhouse (24 ± 5°C, L, D 16:8 h) and watered every other day.

### 2.2 Insects

The banded cucumber beetle (*Diabrotica balteata* (LeConte, 1865, Coleoptera: Chrysomelidae) originates from the tropical Americas (Teng et al., 1984, Moreira et al., 2015, Pitre & Kantack, 1962) and is considered a pest of agricultural crops including beans, sweet potatoes, and cucurbits. Larvae feed belowground on roots, while adults eat leaves and flowers (Pitre & Kantack, 1962, Teng et al., 1984). Eggs used to establish our *in‐house* colony were supplied by Syngenta (Stein, Switzerland). Emerging larvae were maintained in soil (Sinntal‐Altengronau, Deutschland), fed on roots of 4‐day‐old maize seedlings (Hybrid DFI 45321, DEFI genetics AG, Switzerland), and kept at 25 ± 2°C, 60% RH, and 16:8 h L/D cycles. First and second instar larvae were used in all experiments.

The rove beetle *Dalotia coriaria* Kraatz, Coleoptera: Staphylinidae (formerly *Atheta coriaria*, [Gusarov, 2003]), is a soil‐dwelling predator used as a biological control agent for certain greenhouse pests (Birken and Cloyd, 2007). Both larvae and adults feed on various arthropod pests (Helyer et al., 2003). It is a generalist predator that did not coevolve with chrysomelids and is not expected to be adapted to the potentially deterrent and toxic effects of cucurbitacins (Miller and Williams, 1983). Eggs were purchased from Andermatt Biocontrol (AG, Switzerland) and a laboratory rearing was established. Predators were kept in controlled conditions (24°C, 60% R.H. and L:D 16:8 h) with coconut fiber and vermiculite as a substrate and fed on oat and dog pellets. Adults of *D. coriaria* were used in all experiments.

### 2.3 Cucurbitacin content in roots of wild and domesticated plants and in larvae of *D. balteata*

To verify the cucurbitacin content in the squash roots of wild accessions and domesticated varieties and in the larvae that fed on these roots, we quantified the cucurbitacin content in the roots of the different plants (*N* = 4 plants/host plant treatment) and in the larvae of *D. balteata* (*N* = 3 per time point) fed on roots of either wild or domesticated plants. The cucurbitacin content in the larvae was measured with dead larvae, because the feeding preference bioassays were performed with frozen larvae.

Roots of 2‐week‐old plants of *C. argyrosperma* were harvested, immediately flash frozen and ground to a fine powder in liquid nitrogen. Then 100 mg of sample powder was weighed with a microbalance (Mettler Toledo XP&, Columbus, Ohio, USA) and put in a 1.5‐ml Eppendorf tube. We added 1 ml of MeOH 100% and five glass beads per tube.

Larvae of *D. balteata* fed on roots for 7 days were collected and immediately frozen at −80°C. Larvae were taken out of the freezer and we quantified the content of cucurbitacins in the larval body 2 h and 24 h later. Twenty mg (12 larvae in average) per sample was weighed with a microbalance (Mettler Toledo XP&) and put in a 1.5‐ml Eppendorf tube to which 250 μL of MeOH 100% was added. The samples were ground with a pellet pestle, then another 250 μL of MeOH 100% were added (total of 500 μL in the tube per sample).

All samples (roots and larvae) were vortexed and centrifuged at 4°C (10 min, 9000 rpm). The supernatants from the larvae samples were filtered (13 mm Syringe filter, PTFE hydrophilic, 0.22 μm, BGB, CHE) before the analysis. Supernatants were used for analysis by liquid chromatography‐mass spectrometry as described by Jaccard et al. (2021).

Briefly, the analysis of cucurbitacins was performed by UHPLC‐QTOFMS using an Acquity UPLC™ coupled to a Synapt G2 high‐resolution mass spectrometer (Waters). The column used for chromatography was an Acquity UPLC BEH C18 1.7 μm, 2.1 × 50 mmm (Waters). Data acquisition was performed with the software Masslynx™ v.4.1 (Waters). Cucurbitacins were identified based on their molecular formula and fragmentation patterns provided by accurate mass measurements, and using available databases such as the Dictionary of Natural Product (CRC Press). In some cases, the presence of several possible cucurbitacin isomers prevented full identification. Peaks corresponding to known cucurbitacins were automatically integrated using TargetLynx XS™ with a 0.1‐min chromatographic window centered on the retention time of each component and a 0.02‐Da mass window centered on the (M + HCOO) ion. Quantification of all cucurbitacins was done by external calibration using cucurbitacin B solutions at 0.02, 0.08, 0.4, 2, 5, and 10 μg/ml The cucurbitacin concentration was expressed in μg per g of fresh plant or larva material.

### 2.4 Preferences of *D. coriaria* for *D. balteata* larvae fed on different wild populations and cultivated varieties of squash

With this experiment, we tested the hypothesis that predators will preferentially select *D. balteata* larvae that were fed on the low‐cucurbitacin plants. For this, we evaluated the preference of *D. coriaria* adults for *D. balteata* larvae fed on roots either of wild populations or domesticated varieties in a choice test. Second instar larvae were fed on high‐cucurbitacin roots of two wild populations of *C. argyrosperma* (WB and WU) and no‐cucurbitacin roots of four domesticated varieties (FVP, FSE, OCT, and ONC) for 7 days. Then, larvae were frozen at −80°C. The predation assay was performed with dead‐frozen larvae to allow us to standardize the size of the larvae and distinguish the high‐cucurbitacin larvae versus no‐cucurbitacin larvae in their marked spot in the Petri dish. The experiment was performed in a red Petri dish to simulate darkness (as in the soil) (Sarstedt, Ø 8 cm). To maintain humidity, 850 μL of water was added on the filter paper placed in the Petri dish. Adults were starved for 12 h prior to use. One *D. balteata* larva fed on roots of wild squash (either WB or WU) with cucurbitacins was placed on one side on the Petri dish, and on the opposite side (approximatively 7 cm distant from each other), one larva fed with no‐cucurbitacin roots (either FSE, FVP, OCT, or ONC). One adult rove beetle was released in the center of the arena. Petri dishes were sealed with parafilm to prevent the predator from escaping. A total of eight combinations were performed, always one high‐cucurbitacin larva against one no‐cucurbitacin larva: WB × ONC, WB × OCT, WB × FSE, WB × FVP, WU × ONC, WU × OCT, WU × FSE, WU × FVP with 20 replicates per combination and the experiment was performed twice. The predation rate was recorded after 24 h. A predation event was recorded when the hemolymph of the larva was found leaking from the body or when body parts were removed (Figure S1). Larvae without any signs of damage/attack were considered not predated. As comparison (control) for the predation damage/attack, to account for natural deterioration of the dead larvae, we also had five Petri dishes without a predator.

### 2.5 Predator survival on *D. balteata* larvae fed on different wild populations and cultivated varieties of squash

We tested the hypothesis that cucurbitacins sequestered by *D. balteata* larvae can be deleterious for the predators. Thus, we evaluated *D. coriaria* survival after they were fed on dead *D. balteata* larvae that had been reared on wild populations (high‐cucurbitacin roots) or on domesticated varieties (no‐cucurbitacin roots) of *C. argyrosperma* for 7 days. The predators were individually placed in cells of a tray (each with 32 cells) and fed with one of seven diet treatments (*n* = 32; see Supplementary Figure 2): *D. balteata* larvae that fed on either roots of two wild populations (WB and WU) or roots of four domesticated varieties (FVP, FSE, OCT, and ONC) and one control diet consisting of frozen eggs of *Ephestia sp*. (Pyralidae) (Andermatt Biocontrol, Switzerland). Transparent stickers were used to cover the top of each cell to prevent predator escape. We added a moist filter paper on the bottom of each cell as a source of water and to maintain humidity. For 7 days, every 24 h, predator survival was evaluated and the diet was replaced. The trays were kept in the dark to simulate soil darkness. If a predator escaped the cell, the replicate was removed from the analysis (between one to five replicates were lost per treatment).

### 2.6 Performance and survival of *D. balteata* larvae on roots of wild populations and cultivated varieties in the presence of a predator

In the previous experiments the predators were exposed to *D. balteata* larvae after they fed on the different plant treatments. In this experiment, we wanted to examine the effect of the plant on the predator’s feeding behavior and survival under more realistic conditions. Thus, larvae were exposed to the predator while still feeding on the plant. We tested two different hypotheses. The first hypothesis was that larvae reared on roots of wild populations will suffer lower predation than larvae fed on domesticated varieties, and the second hypothesis was that predator survival will be higher when offered larvae fed with roots of domesticated varieties with lower cucurbitacin content. In contrast, to the previous experiments, here we used living larvae. We carefully placed the root system of 2‐week‐old squash plants (*n* = 20, Figure S3) from all plants (two wild populations and four domesticated varieties) into a plastic bag. The plants were left for 3 days under laboratory conditions (L: D 16:8 h) to acclimate to the new environment. Then, we added 10, second instar larvae of *D. balteata* in each plastic bag and allowed them to move and feed freely on the roots. Bags were closed under the cotyledons with elastic film (Parafilm; Pechiney Plastic Packaging; Menasha) to prevent larvae from escaping (Figure S2). Larvae were left in the bags for 5 days, then on day 6, we added 20 predators (*D. coriaria*) in half of the bags of each plant treatment. The bags without predators were used as a control for survival and growth of *D. balteata* in the absence of predators. Bags were maintained in the lab at room temperature (22°C ± 2°C) and natural light conditions (L: D 16:8 h) for 4 days. At the end of this period, the bags were opened inside an insect rearing cage to prevent the predators from escaping. The presence and number of both *D. balteata* larvae and predators were recorded. The 4‐day period was chosen to allow enough time for predation and before the pupation of *D. balteata* larvae.

To calculate *D. balteata* larval relative growth rate (RGR), the total weight of the 10 larvae was recorded at the start of the experiment and then 9 days later. For both time points, the individual mean larval weight was calculated by dividing the sum of the weight of the initial (start of the experiment) or recovered larvae (end of the experiment) by the total number of larvae present.

### 2.7 Statistical analyses

Statistical tests were carried out with Sigma Plot software (v. 11; Systat Software Inc.) and R statistical software (v. 4.0.0; R Development Core Team, 2020) and its complementary console R‐studi, using Analysis of Deviance (ANODEV; a maximum likelihood equivalent of ANOVA), followed by residual analysis to verify suitability of distributions of the tested models (Shapiro–Wilk test). We installed the extra R packages “lme4”, “lmerTest”, and “emmeans” to analyze our data.

Krustal–Wallis test followed by a pairwise comparison using Wilcoxon rank sum test with continuity correction were used to analyze the total amount of cucurbitacin content in the plant’s roots. The cucurbitacin concentration in larval bodies was first log transformed before the analysis and then analyzed with a Linear Model (*lm*) followed by least squares means (*LSMeans*) to compare among host plant food source (wild populations vs. domesticated varieties) for the two different time points. A partial least squares discriminant analyses (PLS‐DA) and hierarchical clustering heatmap were carried using MetaboAnalyst 5.0 to check for differences in cucurbitacin profile among wild population of *C. argyrosperma* and *D. balteata* larvae.

Predator feeding preference (choice‐test) was analyzed for each treatment combination with a generalized linear model (*glm*) with binomial distribution. Least Squares Means (*LSMeans*) were used to compare differences among treatments.

The survival of the predator on *D. balteata* larvae previously fed on different squash roots and *Ephestia* eggs (control) was analyzed using Kaplan–Meier estimator by log‐rank method with the Sigma Plot software.

Relative growth rate (RGR) and survival rate of *D. balteata* larvae in the presence or absence of the predator was first log‐transformed, then analyzed with a linear model (LM) with variety/population as explanatory variables followed by least squares means (*LSMeans)* to compare among host plant treatments. The RGR and survival rate differences among larvae fed on wild squash plants and domesticated varieties selected for different purposes (consumption or ornamental) was analyzed with a generalized linear mixed model (*lmer*) with purpose of domestication as the explanatory variable and wild populations and domesticated varieties as random factors.

## 3 RESULTS

### 3.1 Cucurbitacin content in roots of wild and domesticated plants and in larvae of *D. balteata*

**FIGURE 1:**
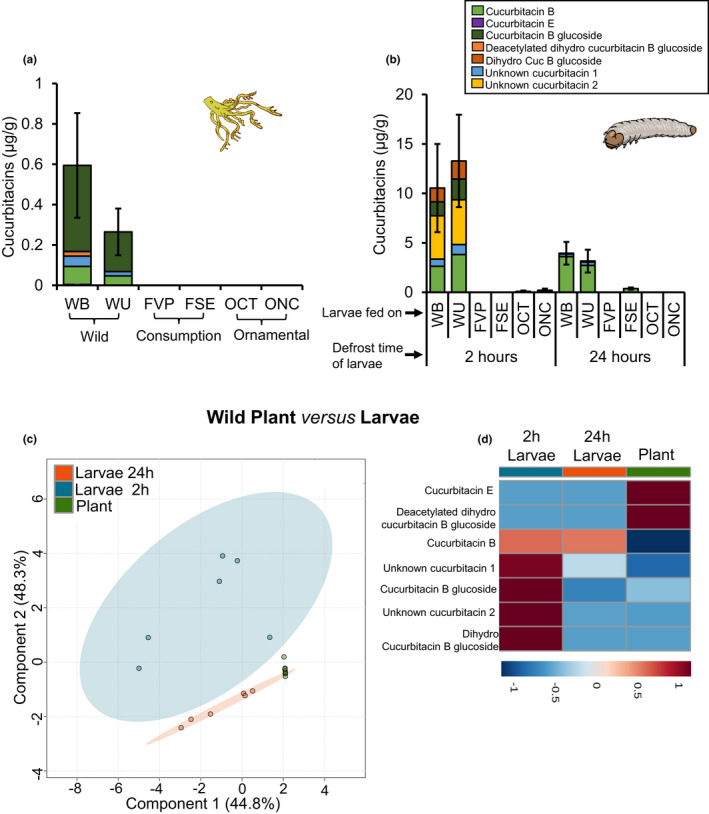
Differences in cucurbitacins profiled in *C. argyrosperma* roots and *D. balteata* larvae. (a) Cucurbitacin levels in squash roots (*n* = 4) of 2‐week‐old plants of wild populations (WU and WB), consumption (FVP and FSE) and ornamental varieties (OCT and ONC). (b) Cucurbitacin content in 2 and 24 h defrosted larvae (*n* = 3) that fed on roots of wild plants, consumption and ornamental varieties for 7 days. Bars represent the mean (±SE). (c) Results of a discriminant analysis (PLS‐DA) and (d) hierarchical clustering heatmaps of the cucurbitacins present in roots of squash and larvae that fed on these roots. Different letters indicate significant differences between treatments within each time point (*p* values are given for treatments [generalized linear model (family, Gaussian)] followed by pairwise comparisons of least squares means (LSMeans). **p < 0.01, ***p < 0.001

The total amount of cucurbitacins in the roots were significantly different between wild populations and domesticated varieties (*χ* ^2^_[5]_ = 26.805, *p* = 0.0001, Figure 1a). Roots of all four domesticated varieties, did not contain any cucurbitacins, whereas the roots of the two wild populations contained high concentrations of cucurbitacins (more than 10 μg/g). No significant differences in cucurbitacin content were found between the two wild populations (WB‐WU: *p* = 0.264, Figure 1a).

In accordance with the cucurbitacin content found in the plants, we found that 2 h after defrosting, larvae that had fed on roots of wild plants contained cucurbitacins in their body, while larvae that had fed on the domesticated varieties did not (*F*_3,12_ = 10.61, *p* = 0.001; Figure 1b). No significant difference was found in cucurbitacin content between larvae that had fed on roots of the two wild populations (*t* = 0.615, *p* = 0.925, Figure 1b). After 24 h outside the freezer, the cucurbitacin content in the larvae drastically decreased and the difference between larvae fed on wild plants and domesticated varieties was less evident (*F*_3,12_ = 3.481, *p* = 0.038). Results clearly show that the larvae actively sequester and accumulate the cucurbitacins, since the levels in their body were much higher compared to the amount found in the roots (Figure 1a,b). Interestingly, the cucurbitacin profiles differed between the roots and the larvae that fed on these roots (Figure 1c,d). Two cucurbitacins (Cucurbitacin E and deacetylated dihydro cucurbitacin B glucoside (or isomer) were exclusively found in the roots (Table S1).

In the 2 h‐defrosted larvae we found in total five compounds, but after 24 h only three. The main differences observed between roots and larvae were after 2 h, the larvae had high amounts of an isomer of a dihydro form of Cuc B glucoside and an unknown Cuc (Figure 1a–d). However, these compounds disappeared after 24 h. This difference could be due to either degradation or transformation during the defrosting process.

### 3.2 Cucurbitacin content in *D. balteata* larvae does not affect the feeding preference of *D. coriaria*

**FIGURE 2:**
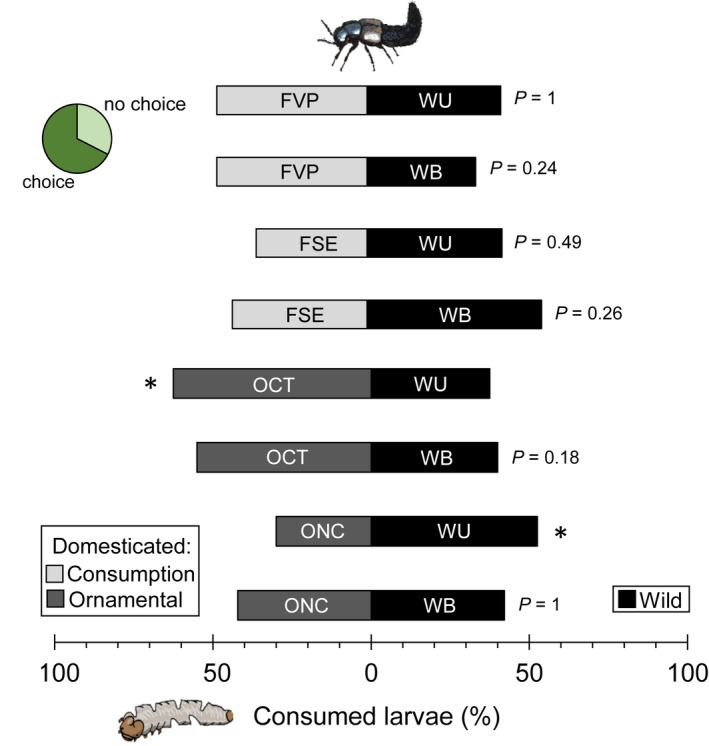
Predation preference by *D. coriaria* for *D. balteata* larvae that fed on roots of wild populations (*n* = 40, WU and WB = black) with cucurbitacins and larvae that fed on domesticated varieties (fruit consumption: FVP and FSE and ornamental: OCT and ONC, shades of gray, *n* = 40) without cucurbitacins. Feeding preference was evaluated after 24 h. Bars are the percentages of beaten larvae. Pie chart indicate the overall proportion of predators that ate or not during the assay for all treatment combine. Bonferroni corrected *p* values are given for treatment comparisons [generalized linear model (family, Binomial)], followed by pairwise comparisons of least squares means (LSM). *p < 0.05

The predator *D. coriaria* did not distinguish between *D. balteata* larvae that had fed on high‐cucurbitacin roots (WB and WU) or on no‐cucurbitacin roots (FSE, FVP, OCT, and ONC) (Figure 2). The predators were apparently not deterred by the cucurbitacins present in the larvae. Predator preference was recorded as significant for two of the combinations, both include an ornamental variety and the wild population WU. Nevertheless, the preference appears not to be linked to cucurbitacin‐content, as in one case predators significantly chose larvae fed with domesticated roots (OCT, no‐cucurbitacin roots) over the larvae that ate wild squash roots (WU) (*p* = 0.04) and in the other case they showed the opposite pattern, larvae that fed on wild (WU, high‐cucurbitacin roots) over larvae that fed on domesticated roots (ONC) (*p* = 0.03).

### 3.3 Predator survival is not affected by the content of cucurbitacins in *D. balteata* larvae

**FIGURE 3:**
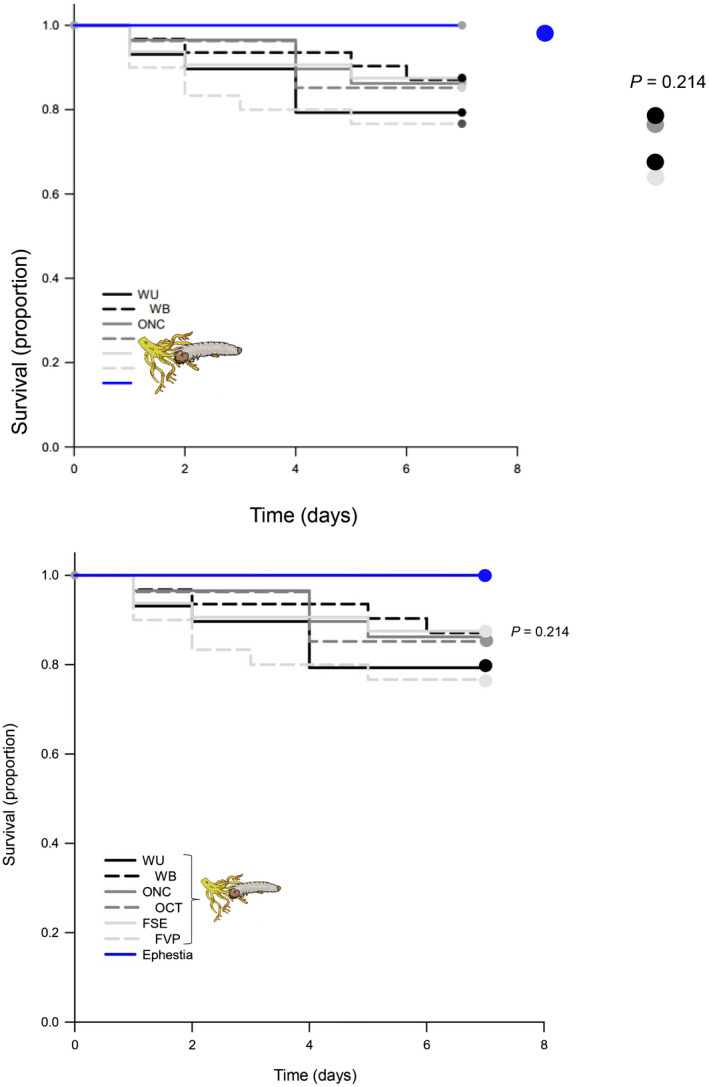
Survival of *D. coriaria* exposed to *D. balteata* larvae fed with roots of wild populations (WU: Black (*n* = 29), WB: Black (*n* = 31)) and domesticated varieties (fruit consumption: FVP (*n* = 30) and FSE (*n* = 32) and ornamental: OCT (*n* = 27) and ONC (*n* = 30), shades of gray) and *Ephestia* eggs used as control (*n* = 30, blue). The y axis indicates the proportion of survived predators. The x axis represents the time in days. Larvae were frozen 2 h after collection from the squash roots and defrosted 30 min before the start of the experiment. The effect of treatment on predator survival was analyzed using Kaplan–Meier estimator by log‐rank method

The survival of the predator (*D. coriaria*) was not affected by the content of cucurbitacins in *D. balteata* larvae fed with roots of wild or domesticated plants (*p* = 0.214; Figure 3). After 7 days, predator survival was between 76% and 93% and no significant differences were found among plant treatments as compared to the control.

### 3.4 Predation of *D. balteata* by *D. coriaria* is affected by larval plant‐diet

The survival of *D. coriaria* was high when placed in the bags with the *D. balteata* larvae feeding on squash roots. Between 18 and 20 predators were consistently recovered from the bags at the end of each experiment. The fact that the predators did eat the larvae of *D. balteata* (Figure S4), indicates that the cucurbitacins present in the roots of wild plants did not affect their survival.

**FIGURE 4:**
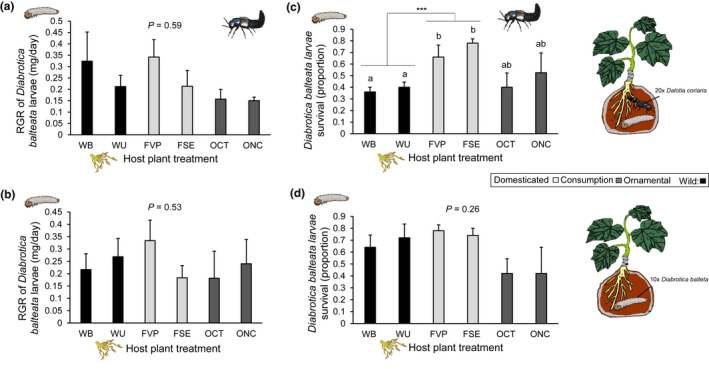
Relative growth rate of *D. balteata* larvae feeding on roots of wild populations of *C. argyrosperma* (WB and WU = black, *n* = 10) or domesticated varieties (FVP and FSE selected for fruit consumption, OCT and ONC selected for an ornamental use, shades of gray, *n* = 10) with (a) and without (b) predators after 9 days. Survival of *D. balteata* larvae with (c) and without (d) predators. Larvae fed on roots of wild populations of *C. argyrosperma* (WB and WU = black, *n* = 10) or with domesticated varieties (FVP and FSE selected for fruit consumption, OCT and ONC selected for an ornamental use, shades of gray, *n* = 10) after 9 days. Bars are RGR (A and B) or the proportion of survived larvae (C and D) (+SE). Letters indicate the difference on herbivore survival among host plant treatments with predator presence. *p*‐values are given for differences among host plant treatments (wild populations and domesticated varieties) [generalized linear model]. *** indicates significant differences between wild populations and domesticated varieties selected for fruit consumption

The RGR of *D. balteata* larvae was not significantly different among wild populations and domesticated varieties with or without predators (with predator: *F*_5,46_ = 0.75, *p* = 0.59, Figure 4a, without predator: (*F*_5,49_ = 0.82, *p* = 0.53, Figure 4b). Similarly, when pooled by domestication status or purpose of domestication (wild, fruit consumption, and ornamental use), we did not find significant differences in larval RGR (*F*_2,49_ = 1.664, *p* = 0.199).

In contrast, predator presence had a significant effect on larval survival across plant treatments (*F*_5,48_ = 4.23, *p* = 0.002, Figure 4c). When pooled by domestication status and purpose, survival of *D. balteata* larvae was lower when feeding on roots of wild populations than on roots of domesticated varieties selected for consumption (*F*_2,51_ = 10.506, *p* = 0.0001, Figure 4c), but not different from larvae feeding on ornamental plants. However, in the absence of predators in the bag, no significant differences in larval survival were found among host plant treatments (*F*_5,50_ = 1.059, *p* = 0.394, Figure 4d) nor among purpose of domestication (*F*_2,53_ = 2.428, *p* = 0.09).

## 4 DISCUSSION

The most important trait selected during the domestication of the genus *Cucurbita* (squash) is the absence of cucurbitacins in the fruits (Navot et al., 1990, Gry, 2006). We confirm here for *C. argyrosperma* that cucurbitacins were also selected out from the roots of domesticated varieties (Figure 1a).

*Diabrotica balteata*, as many other species of Chrysomelidae in the subfamily Galerucinae is capable of sequestering cucurbitacins through the ingestion of plant tissue (Metcalf, 1979, Metcalf, 1986, Metcalf and Lampman, 1989). The assumed reason for this is that sequestered cucurbitacins provide protection against natural enemies (Nishida and Fukami, 1990, Nishida et al., 1992). Because of the lower levels of cucurbitacins in domesticated squash, we hypothesized that *D. balteata* larvae feeding on roots of wild plants would be more protected from predators than larvae feeding on no‐cucurbitacin roots of domesticated varieties. We tested this for the predatory rove beetle *D. coriaria* expecting lower preference for, and survival on, beetle larvae fed with roots of wild plants than when offered larvae that had fed on roots of domesticated varieties.

Our analyses of cucurbitacin content in the larvae showed that they sequester cucurbitacins when feeding on these roots. Yet, contrary to our prediction, larvae of *D. balteata* fed on roots of wild squash were not protected from predation by *D. coriaria*. *D. coriaria* survival was not different when offered beetle larvae fed with high‐cucurbitacin roots (wild populations) or no‐cucurbitacins roots (domesticated varieties). Moreover, in the choice‐test the larvae from the different plant treatments were equally attacked by the predator. The sequestration ability of *D. balteata* larvae was further confirmed in a recent study with bitter and non‐bitter cucumber plants (Bruno et al. 2021, unpublished data) in which it was also found that this does not provide any protection against this and other natural enemies, as detailed below.

Of further interest is the fact that cucurbitacin profiles were different between plants and sequestering larvae (Table S1). *Diabrotica balteata* larvae contained high amounts of an isomer of a dihydro form of Cucurbitacin B glucoside and an unknown cucurbitacin, but these two compounds were not found in the plants. This implies that the cucurbitacins ingested by *D. balteata* larvae are converted into different compounds and then stored in their tissues. These results are in line with those of numerous studies that found that herbivores that sequester plant compounds transform them into different compounds (Trigo et al., 1994, Hartmann et al., 2005). We also found that the concentrations of cucurbitacins in the larvae were much higher than in the roots, indicating true sequestration. At the beginning of the predator survival and choice bioassays, the larvae from wild squash contained more than 10 μg/g cucurbitacins, whereas larvae that had fed on domesticated squash contained barely detectable amounts (Figure 1b). After being defrosted for 24 h, the difference was still evident, but cucurbitacin content had diminished to less than 5 μg/g, most likely due to larval decomposition.

Another unexpected result of this study was that the RGR of *D. balteata* larvae was similar when feeding on roots of wild plants and of domesticated varieties. In a different study using these same and other wild populations and domesticated varieties of *C. argyrosperma*, we found very large differences in RGR rate; *D. balteata* larvae grew slower on wild roots than on roots of domesticated varieties (Jaccard et al., unpublished results). In the current study, treatments consisted of feeding the larvae on plants with high (wild) or no‐cucurbitacin (domesticated) content. It is possible that not only presence or absence, but also small variations in cucurbitacins between cultivars and wild plants, as well as within plant treatments may account for differences between experiments. Recent studies have documented the importance of chemical variation in plant defenses for herbivore and natural enemy performance in unpredictable and nonlinear ways (Pearse et al., 2018, Paul et al., 2021, Hauri et al., 2021). For instance, Hauri et al. (2021) found that the performance of generalist caterpillars of the cabbage looper *Trichoplusia ni* and their interaction with the generalist predator *Podisus maculiventris*, depended not so much on the cultivar’s chemotype but rather on the chemotype mixture. They also found differences between the performance of males and females of *T. ni* when predators were present, suggesting that differences between the sexes in their needs for growth and development could force larger male caterpillars to move more and feed on more chemotypes than smaller females. Future studies on the effects of cucurbitacins on *D. balteata* and its interactions with natural enemies should consider possible differences in the effects on male and females throughout the insects’ development.

Interestingly, when larvae were exposed to the predators under more natural conditions in the soil (bag experiment), predation was higher on larvae feeding on the wild plants. This further supports the findings that the predator was neither deterred, nor harmed by the cucurbitacins present in the herbivore’s body. The higher predation rates on wild‐fed larvae may have something to do with these larvae developing slower and/or being weaker. However, it would be surprising if early development of *D. balteata* larvae on cucurbitacins containing plants is slower, because it is known that cucurbitacins have a phagostimulant effect on *D. balteata* (Eichenseer and Mullin, 1996, Halaweish et al., 1999, Howe et al., 1976, Tallamy and Halaweish, 1993). For example, when pure cucurbitacin B was added to soybean leaves, *Diabrotica* beetles exhibited enhanced feeding on this plant, which it normally rarely attacks (Metcalf et al., 1980). Similarly, Kim and Mullin (2003) found that adding cucurbitacin B (0.2 nmol/disk) on a cellulose disk stimulated feeding by the western corn rootworm (*D. virgifera virgifera*). Lang et al. (2013) found that for another cucurbit specialist, the coccinellid *Epilachna paenulata,* the phagostimulation properties of cucurbitacins were due to only one type of cucurbitacin among 28 tested. *Diabrotica* beetles share similar stimulatory thresholds to various cucurbitacins irrespective of their degree of Cucurbitaceae specialization, with cucurbitacin B believed to be the most phagostimulatory compound (Metcalf et al., 1982, Tallamy et al., 1997). Given that there appear to be no advantages for *D. balteata* larvae to feed on cucurbitacin‐containing roots, it remains unclear why their feeding is stimulated by these compounds.

Based on the inconsistent preference of the predator for larvae from the high or free‐cucurbitacin treatment, their similar survival on the two types of larvae and the higher predation on *D. balteata* larvae from high‐cucurbitacin roots, we can conclusively reject the idea that cucurbitacins provide protection against this predator. Evidence from earlier studies corroborates this conclusion (Ferguson and Metcalf, 1985, Tallamy et al., 1998, Metcalf, 1986). Moreover, it has never been shown that cucurbitacins have a lethal effect on any invertebrate and the only indication of their toxicity is the increased mortality in some adult chrysomelids when consuming these compounds at high doses and in vertebrates such as, cows and sheep (Metcalf, 1979, Metcalf, 1986, Metcalf and Lampman, 1989, Breyer‐Brandwijk, 1962). Thus, to date the earlier study by Ferguson and Metcalf (1985) is the only one showing a negative effect of herbivore‐sequestered cucurbitacins on a predator. In a recent study we tested a wide range of natural enemies including entomopathogenic nematodes, insect predators (also *D. coriaria*), and pathogens (fungi and bacteria) and found that sequestered‐cucurbitacins by *D. balteata* larvae fed on bitter cucumber varieties did not provide any protection against any of these natural enemies (Bruno et al. 2021, unpublished data). It is therefore increasingly evident, that this commonly assumed protection is not the reason for *Diabrotica* larvae to preferentially feed on Cucurbitaceae and sequester cucurbitacins.

In conclusion, our results show that for *C. argyrosperma*, the dramatic reduction of cucurbitacins as a result of domestication, does not increase the susceptibility of root‐feeding larvae to predatory rove beetles. Instead, larvae feeding on high‐cucurbitacin roots suffered higher predation. Thus, our results do not support the common assumption that sequestered cucurbitacins confer protection against natural enemies. This logically leads to the rejection of our hypothesis that squash domestication has had a positive effect on the third trophic level. The effects of domestication on the third trophic level, particularly pertaining belowground organisms, have been largely neglected. These types of studies can help to identify specific plant traits that could enhance direct and indirect crop resistance to insect pests. Moreover, the fact that *D. coriaria* is not negatively affected by cucurbitacins, could make it a suitable natural enemy in biological control efforts against *D. balteata* and other squash pests.

## CONFLICT OF INTEREST

The authors declare that the research was conducted in the absence of any commercial or financial relationships that could be construed as a potential conflict of interest.

## AUTHOR CONTRIBUTIONS

C.J, C.C.M.A, and B.B. originally formulated the idea. C.J., B.B. C.C.M.A, and N.M. designed the experiments. C.J. and N.M performed all the experiments and wrote the first version of the manuscript. G.C and P.B developed the cucurbitacin extraction and analysis method and identified the cucurbitacins. N.M, C.C.M.A, and C.J analyzed the data. N.M did the drawings. All co‐authors contributed to the writing of the last version of the manuscript.

## Supporting information


Supplementary Material S1

## Data Availability

All the data are presented in figures, tables, and Supporting Information.
