# Supplementary material for: The effect of squash domestication on a belowground tritrophic interaction
Source: Plant Environ Interact. 2022 Mar 4;3(1):28–39. doi: 10.1002/pei3.10071 (PMC10168047; doi:10.1002/pei3.10071)
Supplement: Supplementary file 1 — Supplementary Material S1 [file PEI3-3-28-s001.docx]

**Supplementary materials**


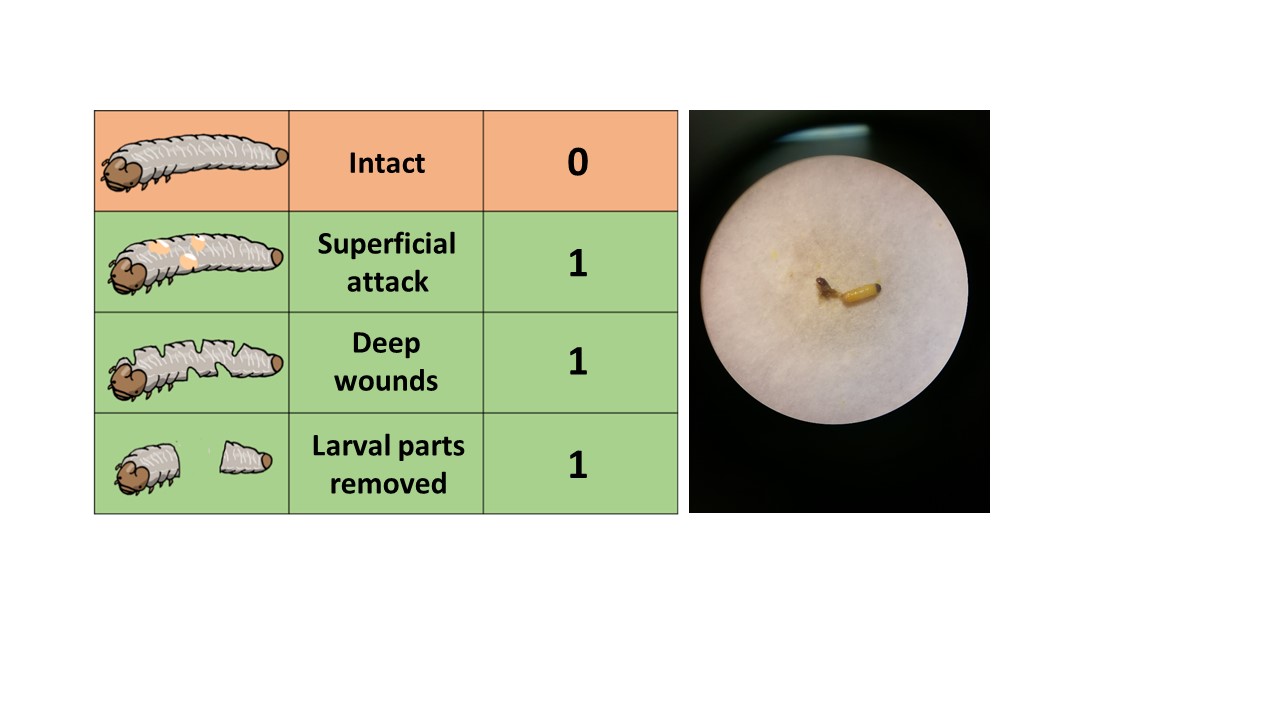


**Figure S1**. Preference indicators. When the type of damage illustrated in the greens squared was observed, the *Diabrotica balteata* larvae were considered as chosen.


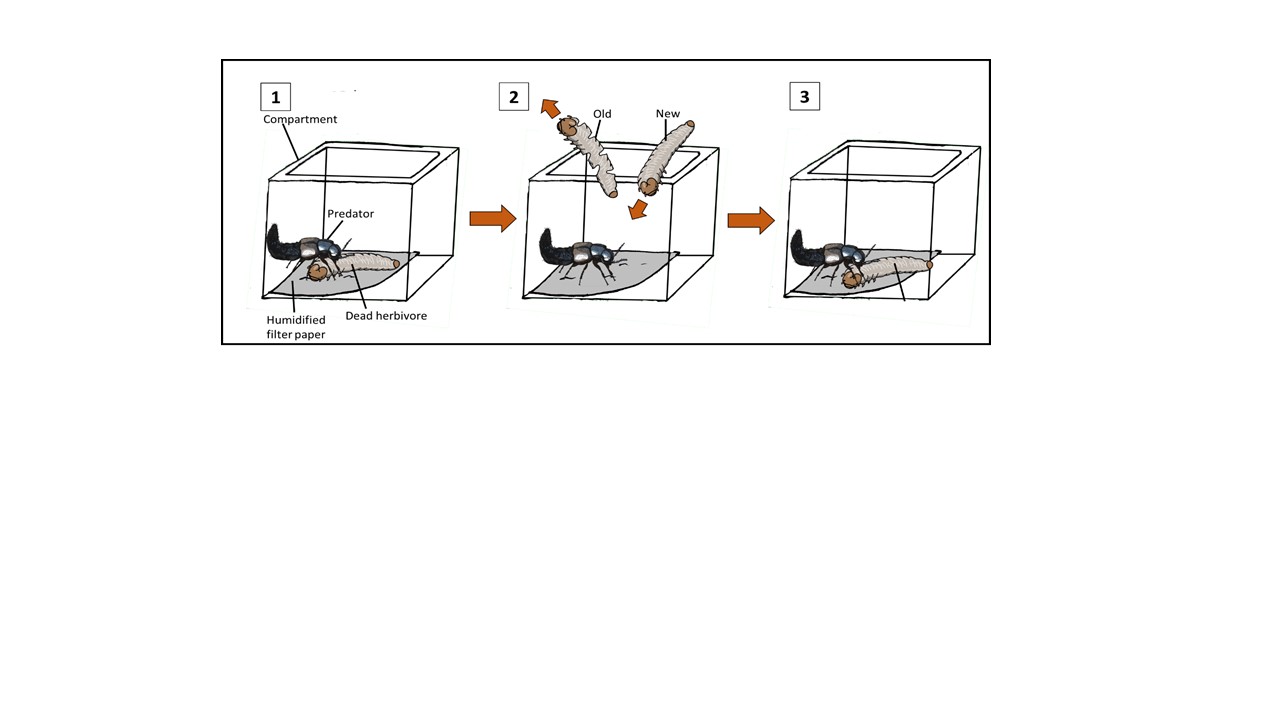


**Figure S2**. *Dalotia coriaria* survival experiment in trays with 32 compartments (cells). One dead *Diabrotica balteata* larvae, one *Dalotia coriaria* were placed in each compartment with humidified paper filter as source of water. The dead larvae were replaced every day to avoid degradation.


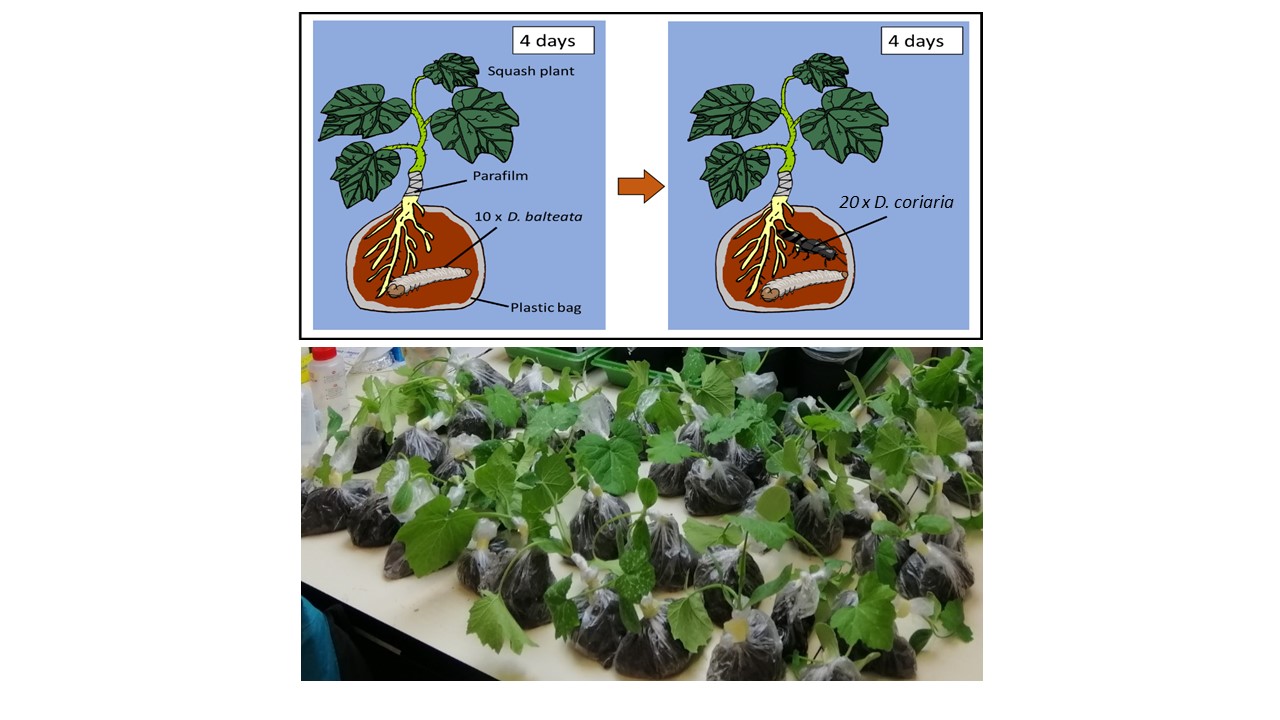


**Figure S3:** Individual squash plant enveloped with plastic bag containing roots, 10 *Diabrotica balteata* larvae and 20 *Dalotia coriaria* soil.

**Table S1:** Putative identification of cucurbitacins found in roots of *C. argyrosperma* and *Diabrotica balteata* larvae. Cucurbitacins were putatively identified based on their exact masses (allowing for molecular formula determination) and retention times and compared with those of the standard Cucurbitacin B as well as with available databases such as the Dictionary of Natural Product (CRC Press).

| CUC | Tissue, Organism | RT (min) | (M+HCOO)^-^ | (M-H)^-^ | MF | PI |
| --- | --- | --- | --- | --- | --- | --- |
| 1 | Roots & larvae | 3.10 | 767.3834 | 721.3779 | C38H58O13 | Dihydrocucurbitacin B glucoside (or isomer) |
| 2 | Roots & larvae | 3.02 | 765.3694 | 719.364 | C38H56O13 | Cucurbitacin B glucoside (or isomer) |
| 3 | Roots & larvae | 3.79 | 603.3174 | 557.2624 | C32H46O8 | Cucurbitacin B |
| 4 | Roots | 4.06 | 601.3016 | 555.2961 | C32H44O8 | Cucurbitacin E (or isomer) |
|  |  |  |  |  |  |  |
| 5 | Roots & Larvae | 2.52 | 723.3651 | 677.3601 | C36H54O12 | Unknown Cucurbitacin 1 |
| 6 | Larvae | 2.16 | 725.3730 | 679.3720 | C36H56O12 | Unknown Cucurbitacin 2 |
| 7 | Roots | 2.95 | 763.3530 | 717.3489 | C38H54O13 | Deacetylated dihydro cucurbitacin B glucoside (or isomer) |

CUC : cucurbitacins, RT : retention time, MF : molecular formula, PI : putative identification


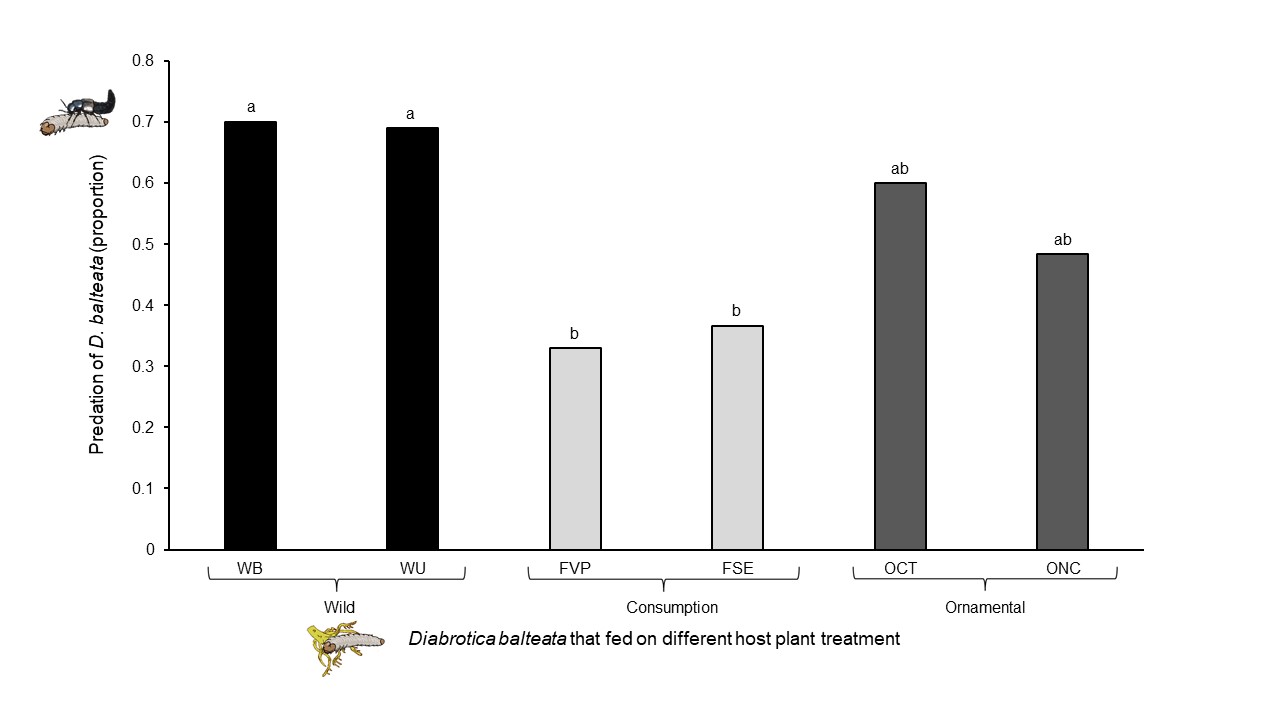


**Figure S4:** Predation of Diabrotica balteata larvae by Dalotia coriaria while herbivore feed on either wild population of Cucurbita argyrosperma (WB and WU in black, n=10) or domesticated varieties (selected for fruit consumption (FVP and FSE in light grey, n=10) and selected as ornamental (OCT and ONC in dark grey, n=10). Bars indicate proportions. Letters indicate predation differences among treatment (P<0.05).
